# Prediction of Functional and Anatomic Progression in Lamellar Macular Holes

**DOI:** 10.1016/j.xops.2024.100529

**Published:** 2024-04-13

**Authors:** Emanuele Crincoli, Barbara Parolini, Fiammetta Catania, Alfonso Savastano, Maria Cristina Savastano, Clara Rizzo, Raphael Kilian, Veronika Matello, Davide Allegrini, Mario R. Romano, Stanislao Rizzo

**Affiliations:** 1 Ophthalmology Unit, Fondazione Policlinico Universitario A. Gemelli IRCCS, Rome, Italy; 2 Eyecare Clinic, Crystal Palace, Brescia, Italy; 3 Department of Biomedical Sciences, Humanitas University, Milan, Italy; 4 Ophthalmology, Department of Surgical, Medical and Molecular Pathology and Critical Care Medicine, University of Pisa, Pisa, Italy; 5 Ophthalmology Unit, University of Verona, Verona, Italy; 6 Catholic University of “Sacro Cuore,” Rome, Italy; 7 Istituto di Neuroscienze, Consiglio Nazionale delle Ricerche, Pisa, Italy

**Keywords:** Lamellar macular hole, OCT angiography, Progression, Biomarkers, Deep learning

## Abstract

**Purpose:**

To use artificial intelligence to identify imaging biomarkers for anatomic and functional progression of lamellar macular hole (LMH) and elaborate a deep learning (DL) model based on OCT and OCT angiography (OCTA) for prediction of visual acuity (VA) loss in untreated LMHs.

**Design:**

Multicentric retrospective observational study.

**Participants:**

Patients aged >18 years diagnosed with idiopathic LMHs with availability of good quality OCT and OCTA acquisitions at baseline and a follow-up >2 years were recruited.

**Methods:**

A DL model based on soft voting of 2 separate models (OCT and OCTA-based respectively) was trained for identification of cases with VA loss >5 ETDRS letters (attributable to LMH progression only) during a 2-year follow-up. Biomarkers of anatomic and functional progression of LMH were evaluated with regression analysis, feature learning (support vector machine [SVM] model), and visualization maps.

**Main Outcome Measures:**

Ellipsoid zone (EZ) damage, volumetric tissue loss (TL), vitreopapillary adhesion (VPA), epiretinal proliferation, central macular thickness (CMT), parafoveal vessel density (VD) and vessel length density (VLD) of retinal capillary plexuses, choriocapillaris (CC), and flow deficit density (FDD).

**Results:**

Functionally progressing LMHs (VA-PROG group, 41/139 eyes [29.5%]) showed higher prevalence of EZ damage, higher volumetric TL, higher prevalence of VPA, lower superficial capillary plexus (SCP), VD and VLD, and higher CC FDD compared with functionally stable LMHs (VA-STABLE group, 98/139 eyes [70.5%]). The DL and SVM models showed 92.5% and 90.5% accuracy, respectively. The best-performing features in the SVM were EZ damage, TL, CC FDD, and parafoveal SCP VD. Epiretinal proliferation and lower CMT were risk factors for anatomic progression only.

**Conclusions:**

Deep learning can accurately predict functional progression of untreated LMHs over 2 years. The use of AI might improve our understanding of the natural course of retinal diseases. The integrity of CC and SCP might play an important role in the progression of LMHs.

**Financial Disclosure(s):**

The authors have no proprietary or commercial interest in any materials discussed in this article.

According to the recent expert consensus^1^ on the topic, what was previously identified as lamellar macular hole (LMH) actually includes 2 separate entities that deserve specific consideration: proper LMH and epiretinal membrane (ERM) foveoschisis. The presence of a contractile ERM, retinal thickening or wrinkling, and the appearance of cystoid spaces in the inner nuclear layer and Henle fiber layer define ERM foveoschisis, which is therefore the new nomenclature used to identify what was previously defined as tractional LMH.^2^ By contrast, proper LMH is characterized by signs of tissue loss (TL) consisting in either the presence of irregular foveal contour (i.e., abnormal, nonlinear shape of the foveal pit contour) or foveal cavity with undermined edges or the presence of ≥1 other sign evoking a loss of foveal tissue (i.e., pseudo-operculum, thinning of the foveal at its center or around). Therefore, when speaking of LMH, we currently refer to what was previously identified in literature as the degenerative type of LMH.^2^ Although anomalous posterior vitreous detachment is believed to be the prime mover of LMH formation,^2^, ^3^, ^4^, ^5^ recent evidence highlighted the pivotal role of parafoveal neurovascular unit integrity in the progression of the disease.^6^^,^^7^ Nevertheless, progression is detected in only around 30% of cases,^8^ and spontaneous resolution has been reported in 5% of cases.^9^ This means on the one hand that a wait and watch strategy could be advisable in most cases and, on the other hand, that refinement of prognostic prediction is required to identify cases that are destinated to progression in case surgical treatment is not performed. Artificial intelligence (AI) has shown promising results in prognostic evaluation of vitreoretinal interface diseases.^10^ Visualization methods in deep learning (DL) have also been used to identify and validate prognostic biomarkers in retinal diseases, helping understand their natural course.^11^^,^^12^ In this study, we aimed to elaborate a DL model for detection of cases of idiopathic LMH at risk of functional progression. The secondary aim was to perform a comprehensive assessment of OCT B-scan and OCT angiography (OCTA) biomarkers of anatomic and functional progression on a large population of LMHs using classic and AI-based methods.

## Materials and Methods

### Design of the Study and Population

This multicentric retrospective observational study was conducted in adherence with the principles of the Declaration of Helsinki and Italian legislation, and it received approval from the ethics committee of the involved centers. Electronic files of patients referring to 3 specialized vitreoretinal centers located in Italy (Policlinico Universitario Agostino Gemelli [Rome], Humanitas Castelli Hospital [Bergamo] and Eyecare Clinic [Brescia]) from January 2014 to May 2023 were screened for the presence of the words “LMH” or “lamellar macular hole.” Exclusion criteria were age <18 years, previous vitreoretinal surgery, previous ocular trauma, diagnosis of macular telangectasia, neovascular age-related macular degeneration, glaucoma, inflammatory eye diseases, high myopia, current or previous treatment with tamoxifene, previous full-thickness macular hole (FTMH), low quality of the images, and clinically significant dioptric media opacity. Cataract was considered as visually significant according to LOCS III criteria (N >3, C >3, P >2).^13^ Only eyes for whom both high-quality (Q score >15) OCT B-scan and OCTA acquisition were available in 2 different timepoints with an interval of 2 years were included. Availability of an OCT B-scan acquisition at 6 months from baseline was also among inclusion criteria. OCT B-scan and OCTA images were acquired with Heidelberg Spectralis HRA + OCT (Heidelberg Eye Explorer, Version 1.10.4.0, Heidelberg Engineering). Required scanning protocol for OCT B-scan and OCTA are detailed in File S1 (available at www.ophthalmologyscience.org).

### Primary Aim of the Study: Development of the DL Model

The primary aim of the study was to elaborate a DL model for prediction of functional progression in LMHs based on OCT B-scan and OCTA acquisitions. Functional progression was defined as a loss of ≥5 ETDRS letters from baseline (date of the first available OCTA acquisition) to follow-up timepoint (24 months after baseline acquisition). Patients were divided in 2 groups according to the presence (visual acuity [VA]-PROG group) or absence (VA-STABLE group) of a functional progression which was not attributable to causes different from LMH evolution during the course of the follow up.^14^ ETDRS VA at baseline examination and after 2 years of follow-up was collected from clinical records. In case ETDRS VA assessment was not available, ETDRS-equivalent letters conversion was performed from Snellen VA according to the following formula:

ETDRS-equivalent letters = 85+ 50 × log(Snellen fraction)^15^

**Figure 1:**
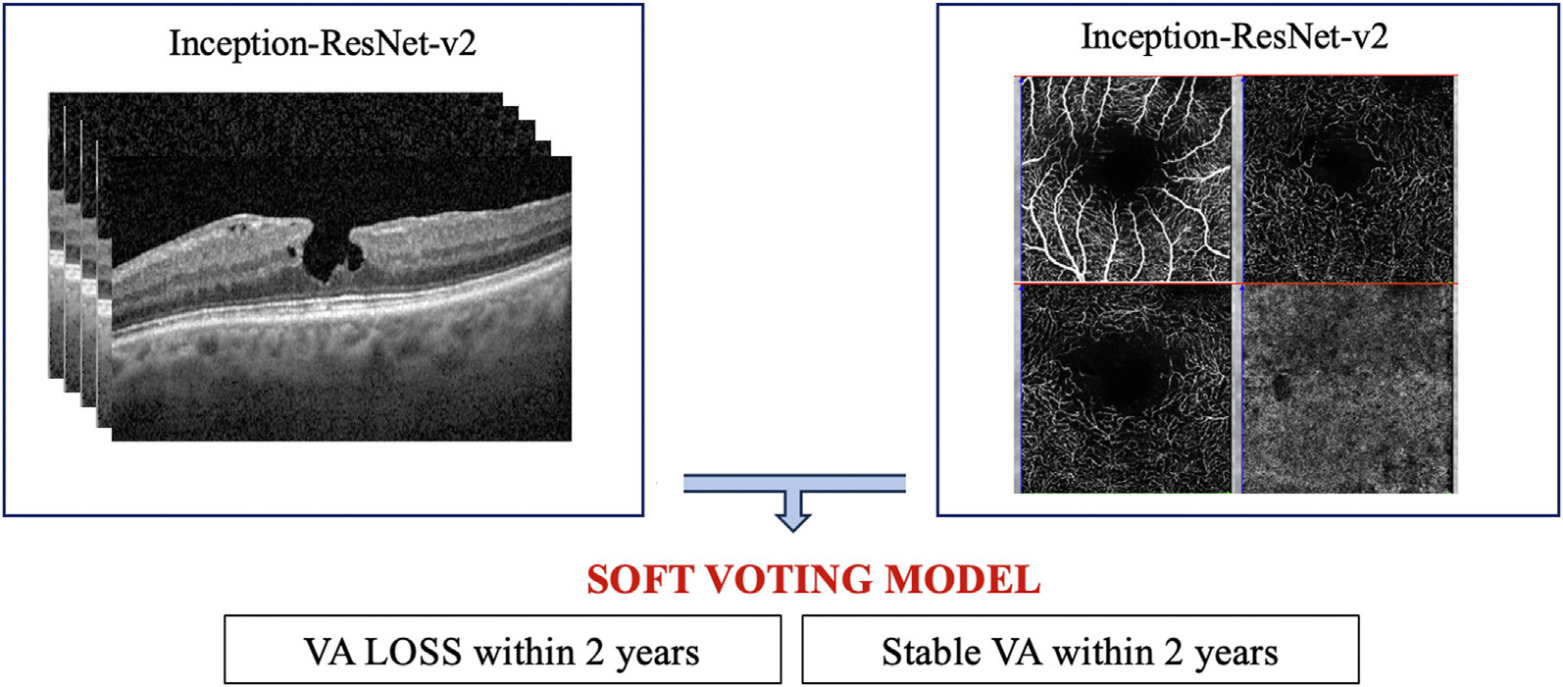
Architecture of the DL model used. Two independent models based on Inception-Resnet-v2 architecture were trained with baseline OCT B-scan and OCTA acquisitions respectively. The OCT B-scan model was based on 5 foveal and perifoveal acquisitions, whereas the OCTA model combined in a multiple input architecture the en face acquisitions of SCP, ICP, DCP, and CC. The output of each model was then combined with a soft voting ensembling technique. With this technique, the degree of certainty of the 2 models for each outcome was combined to take the final decision. In example, if OCT B-scan based model predicted VA progression with a degree of certainty of 60%, and the OCTA model predicted VA stability with a degree of certainty of 70%, the final output was VA stability. CC = choriocapillaris; DCP = deep capillary plexus; DL= deep learning; ICP = intermediate capillary plexus; OCTA = OCT angiography; SCP = superficial capillary plexus; VA = visual acuity.

Matlab (Mathworks) DL toolbox was used as a framework for the DL process. Two independent models (based respectively on OCT B-scan and OCTA images) were trained to distinguish VA-PROG group images from VA-STABLE group images (binary classification). A pretrained version of Inception-ResNet-v2 with introduction of additional dropout layers in node regions (as per a previously described method)^10^ was used as a structure for both models. Models were trained with 70%, validated with 10%, and tested with 20% of available images. Input images were 5 OCT B-scans from each eye (selected as described in the previous paragraph and used as multiple parallel inputs) for the OCT B-scan model and unprocessed images from superficial capillary plexus (SCP), intermediate capillary plexus (ICP), deep capillary plexus (DCP), and choriocapillaris (CC) from each eye (used as multiple parallel inputs) for OCTA model. The 2 architectures were joined with a soft voting node. This technique allows one to take into account the weighted output of both convolutional neural networks (CNNs; Fig 1). Further details on the training settings are available in File S2^16^^,^^17^ (available at www.ophthalmologyscience.org).

### Secondary Aim of the Study: Assessment of Biomarkers of Progression

The secondary aim was to assess biomarkers of functional and anatomic progression using regression analysis (direct method), feature learning, and GradCAM visualization (AI-based methods).^11^ Anatomic progression was defined as a loss of foveal tissue from volumetric reconstruction of baseline acquisition to volumentric reconstruction of follow-up acquisition of >0.01 mm^3^. Functional progression was defined as above. Biomarkers were evaluated on OCT B-scan and OCTA acquisitions.

### Direct Method

The direct method consisted in evaluating the differences between VA-PROG and VA-STABLE groups in terms of OCT B-scan and OCTA variables of interest by means of a binomial logistic regression analysis. Considered OCT B-scan variables included ellipsoid zone (EZ) disruption, epiretinal proliferation, vitreopapillary adhesion (VPA), tridimensional TL during the first 6 months of follow-up, and central macular thickness (CMT). Ellipsoid zone disruption was defined as a loss of continuity of the EZ within the central 1000-μm diameter on baseline OCT B-scan and was assessed as a dichotomous variable.^18^ Epiretinal proliferation was defined as the presence of epiretinal material of homogenous medium reflectivity located above the inner limiting membrane in the foveal and/or parafoveal area according to the definition by Pang et al^19^ and was assessed as a dichotomous variable. Vitreopapillary adhesion was identified on papillary crossing OCT B-scan (described above) in case of evidence of posterior hyaloid attachment to the borders of the optic disc and was assessed as a dichotomous variable.^20^ Central macular thickness was assessed using automatic calculation performed by the OCT software after careful verification of the segmentation boundaries (quantitative variable). For TL assessment, central 42 sections (central 2.5 mm) of the fovea-centered OCT volume scan acquired at baseline and at 6 months follow-up were exported and uploaded on ImageJ (ImageJ software, National Institutes of Health) as image sequences. Image sequences were binarized using the outer nuclear layer luminance threshold as a reference. The outer nuclear layer was chosen because it represents the retinal layer with the lower mean luminance value. The volume reconstruction for baseline and follow-up volume scan was then obtained with using 3D Viewer plug in. The Volume Calculator plug in was used to calculate the volume of the 2 renders. Subtraction of the 2 volume estimations was performed. Conversion from voxels to mm^3^ was performed using Heidelberg caliper as a reference. Tissue loss was defined as a minimum of 0.01 mm^3^ difference between baseline and 6-month follow-up acquisitions. Considered OCTA variables included parafoveal vessel density (VD) and vessel length density (VLD) for SCP and ICP, and DCP, and flow deficit density (FDD) for CC. Image processing for OCTA quantitative variables assessment was performed using ImageJ software. Parafoveal area was segmented as a region of interest for VD and VLD calculation. Vessel density was calculated as the area of white pixels in binarized SCP, ICP, and DCP en face angiograms. Binarization threshold was set to 0.7 ratio. Vessel length density was calculated as the area of white pixels in binarized and skeletonized SCP, ICP, and DCP en face angiograms. Flow deficit density was defined as the percentage of the area of the CC en face acquisition with flow below a given threshold. For FDD assessment, CC en face images were binarized and processed using Phansalkar adaptive threshold method with a radius of 5 as per Chu et al,^21^ which corresponds to 2 folds the normal intercapillary distance measured in healthy age-matched patients minus 1 standard deviation according to current guidelines for CC image processing.

### AI-Based Method

The AI-based method consisted of 2 distinct strategies:

(1) Visualization of GradCam maps: hot regions were analyzed to identify imaging characteristics that were evaluated as crucial to classification from the DL model.
(2) Support vector machine (SVM) model: 2 separate SVM models were created for automatic detection of cases of anatomic and functional progression. Similar to the DL model, the models were trained on 80% of cases and tested on 20% of cases. The performance of the SVM based on the selected features was considered as a proof of validity of the features as biomarkers. Moreover, the weight of each feature in the model was also considered as an element suggesting the relevance of each feature as a biomarker.

### Statistical Analysis

Statistical analysis was conducted using SPSS software v.26 (IBM). The normality of the distribution for quantitative variables was assessed using the Shapiro–Wilk test. Quantitative variables were described using mean and standard deviation. Univariate comparison between groups was performed using 2-tailed T tests for independent samples. Qualitative variables were compared using the chi-square or Fisher exact test when appropriate. For post hoc analysis in case of multiple comparisons, the Tukey test was chosen. Variables showing statistically significant differences at univariate analysis were included in binomial logistic regression. Performance of the DL model was described reporting accuracy, sensitivity, and specificity.

## Results

A total of 139 eyes of 139 patients with a mean follow up of 2.8 ± 0.7 years were included. Among them, 41/139 (29.5%) eyes belonged to VA-PROG group, and 98/139 (70.5%) eyes belonged to VA-STABLE group. No significant differences in terms of male sex prevalence (*P* = 0.40) or follow-up duration (*P* = 0.89) at baseline were detected between the 2 groups (Table 1). The mean age in VA-PROG group was 70.1 ± 7.2 years, whereas the mean age in the VA-STABLE group was 64.8 ± 6.9 years (*P* = 0.03). Baseline best-corrected visual acuity (BCVA) was significantly lower in the VA-PROG than in the VA-STABLE group, with a mean value of 78.1 ± 2.5 letters and 83.1 ± 1.8 letters, respectively. Best-corrected visual acuity at the end of the follow up was 73.4 ± 1.6 letters in the VA-PROG group and 81.9 ± 1.8 letters in the VA-STABLE group.

**Table 1:** Results of Univariate and Regression Analysis Comparing OCT and OCTA Variables of Interest in Eyes that Encountered VA Progression (VA-PROG group) during the follow-up compared with eyes experiencing VA stability during the follow-up

	VA PROG Group (41 Eyes)	VA STABLE Group (98 Eyes)	*P* Univariate	*P* Regression	OR (95% CI)
Age (yrs)	70.1 ± 7.2	64.8 ± 6.9	**0.03**	**0.03**	**2.5 (2.0–3.2)**
Sex (male)	16/41 (39.0%)	31/98 (31.6%)	0.40		
Follow-up duration (yrs)	2.7 ± 0.7	2.8 ± 0.8	0.89		
BCVA at baseline (ETDRS letters)	78.1 ± 2.5	83.1 ± 1.8	**0.04**	0.13	
Final BCVA (ETDRS letters)	73.4 ± 1.6	81.9 ± 1.8	**< 0.01**	**-**	
Spherical equivalent (D)	−1.37 ± 2.2	−1.02 ± 2.0	0.74		
Baseline OCT B-scan variables					
EZ interruption	13/41 (31.7%)	12/98 (12.2%)	**< 0.01**	**< 0.01**	**3.6 (3.2–4.1)**
LHEP	10/41 (24.4%)	17/98 (17.3%)	0.33		
Vitreopapillary adhesion	16/41 (39.0%)	19/98 (19.4%)	**0.04**	**0.04**	**1.3 (1.1–1.6)**
Tissue loss (mm^3^)	0.03	0.006	**< 0.001**	**< 0.001**	**4.0 (3.7–4.2)**
CMT (μm)	132.4 ± 28.3	157.4 ± 30.1	**0.04**	0.23	
Baseline OCTA variables					
CFZ area (mm^2^)	0.43 ± 0.10	0.39 ± 0.13	0.08		
CFZ circularity	0.52 ± 0.07	0.57 ± 0.08	0.07		
Parafoveal SCP VD (%)	45.1 ± 3.10	50.2 ± 3.26	**< 0.01**	**< 0.01**	**4.2 (3.8–4.5)**
Parafoveal SCP VLD (%)	4.20 ± 0.28	4.44 ± 0.26	**0.03**	**0.04**	**1.8 (1.2–2.4)**
Parafoveal ICP VD (%)	44.3 ± 2.91	49.0 ± 3.02	**0.02**	**0.03**	**2.7 (2.3–3.4)**
Parafoveal ICP VLD (%)	4.70 ± 0.21	5.01 ± 0.25	**0.04**	0.34	
Parafoveal DCP VD (%)	43.0 ± 2.86	48.7 ± 3.50	**0.04**	0.15	
Parafoveal DCP VLD (%)	4.68 ± 0.25	5.01 ± 0.26	0.12		
Choriocapillaris FDD	41.81 ± 7.4	33.62 ± 6.8	**< 0.01**	**< 0.01**	**4.5 (3.7–4.8)**

BCVA = best-corrected visual acuity; CFZ = capillary free zone; CI = confidence interval; CMT = central macular thickness; D = diopters; DCP = deep capillary plexus; EZ = ellipsoid zone; FDD = flow deficit density; ICP = intermediate capillary plexus; LHEP = lamellar hole associated epiretinal proliferation; OCTA = OCT angiography; OR = odds ratio; SCP = superficial capillary plexus; VD = vessel density; VLD = vessel length density.

Statistically significant values are highlighted in bold.

As concerns the primary outcome, the DL model was trained with 487 images (70% of a total of 695 images) and 390 OCT B-scan images (70% of a total of 556 images). Testing was performed over 140 OCT B-scan images and 112 OCTA images (28 eyes). The model showed an accuracy of 92.5%, with a sensitivity of 90.0% and a specificity of 94.1%. The SVM model showed an accuracy of 90.5%. The best-performing features in the SVM model were EZ interruption, TL, CC FDD, and parafoveal SCP VD.

As concerns the secondary outcome, EZ interruption was significantly more prevalent at baseline in the VA-PROG group (13/41; 31.7%) compared with the VA-STABLE group (12/98; 12.2%; *P* < 0.001). Tissue loss was significantly higher at baseline in the VA-PROG group compared with the VA-STABLE group (*P* < 0.001). Among OCT B-scan variables, other significant differences included VPA, whose prevalence was 39.0% in the VA-PROG group and 19.4% in the VA-STABLE group (*P* = 0.04, Table 1), and the lower CMT detected in the VA-PROG group (*P* = 0.04; Table 1). Parafoveal SCP VD and VLD at baseline were significantly lower in the VA-PROG group (*P* < 0.01 and *P* = 0.04, respectively). Similarly, parafoveal ICP VD and VLD were significantly lower in the VA-PROG group (*P* = 0.02 and *P* = 0.04, respectively). Parafoveal DCP VD was also lower in the VA-PROG group (*P* = 0.04). Lastly, CC FDD was significantly higher in the VA-PROG group (*P* < 0.01). Binomial logistic regression resulted in a statistically significant model (*P* < 0.001). The model explained 84.0% of the variance (Nagelkerke R2) with a correct classification probability of 88.0%. Final BCVA was excluded from the logistic analysis due to strong biased correlation with the outcome. Variables significantly correlated with the visual outcome were age (OR, 2.5; 95% confidence interval [CI], 2.0–3.2), EZ interruption (OR, 3.6; 95% CI, 3.2–4.1), TL (OR, 4.0; 95% CI, 3.7–4.2), VPA (OR, 1.3; 95% CI, 1.1–1.6), parafoveal SCP VD (OR, 4.2; 95% CI, 3.8–4.5), parafoveal SCP VLD (OR, 1.8; 95% CI, 1.2–2.4), parafoveal ICP VD (OR, 2.7; 95% CI, 2.3–3.4), and CC FDD (OR, 4.5; 95% CI, 3.7–4.8).

Anatomic progression was detected in 50 eyes (50/139; 35.9%), which formed the AN-PROG group. The remaining 89 eyes (64.0%) formed the AN-STABLE group. Binomial logistic regression model was statistically significant (*P* < 0.001) and explained 88.7% of the variance, with a correct classification probability of 90.1%. OCT B-scan variables that were statistically correlated with anatomic progression in regression analysis included EZ interruption (OR, 3.5; 95% CI, 3.1–3.8), epiretinal proliferation at baseline (OR, 1.9; 1.6–2.2), VPA (OR, 2.1; 95% CI, 1.8–2.4), and CMT (OR, 1.8; 95% CI, 1.6–2.0; Table 2). As concerns OCTA variables, a significant correlation with anatomic progression was found for parafoveal SCP VD (OR, 2.9; 95% CI, 2.6–3.3), parafoveal SCP VLD (OR, 1.7; 1.3–2.3), parafoveal ICP VD (OR, 2.1; 95% CI, 1.8–2.4), and CC FDD (OR, 4.7; 95% CI, 4.2–5.0). Lastly, age (OR, 1.8; 1.6–2.1) and baseline BCVA (OR, 2.3; 95% CI, 1.9–2.7) were significantly correlated with anatomic progression. The SVMs trained to predict functional and anatomic progression based on the study variables reached an accuracy of 90.4% and 92.3% respectively, with the most relevant variables being EZ interruption, vitreopapillary adhesion, parafoveal SCP VD, and CC FDD.

**Table 2:** Results of Univariate and Regression Analysis Comparing OCT and OCTA Variables of Interest in Eyes that Encountered Anatomic Progression (AN-PROG group) during the Follow-Up Compared with Eyes Experiencing Anatomic Stability during the Follow-Up

	AN PROG Group (50 Eyes)	AN STABLE Group (89 eyes)	*P* Univariate	*P* Regression	OR (95% CI)
Age (yrs)	69.7 ± 7.0	65.9 ± 7.1	**0.04**	**0.04**	**1.8 (1.6–2.1)**
Sex (male)	19/50 (38.0%)	28/89 (31.5%)	0.43		
Follow-up duration (yrs)	2.8 ± 0.6	2.8 ± 0.7	0.99		
BCVA at baseline (ETDRS letters)	77.2 ± 2.3	83.8 ± 1.1	**0.03**	**0.02**	**2.3 (1.9–2.7)**
Final BCVA (ETDRS letters)	74.9 ± 1.8	82.0 ± 1.5	**< 0.01**	**-**	
Spherical equivalent (D)	−1.15 ± 2.1	−1.26 ± 2.4	0.81		
Baseline OCT B-scan variables					
EZ interruption	15/50 (30.0%)	10/89 (11.2%)	**< 0.01**	**< 0.01**	**3.5 (3.1−3.8)**
LHEP	15/50 (30.0%)	12/89 (13.5%)	**0.02**	**0.03**	**1.9 (1.6–2.2)**
Vitreopapillary adhesion	19/50 (38.0%)	16/89 (18.0%)	**< 0.01**	**0.02**	**2.1 (1.8–2.4)**
CMT (μm)	126.5 ± 26.9	163.2 ± 28.5	**0.02**	**0.02**	**1.8 (1.6–2.0)**
Baseline OCTA variables					
CFZ area (mm^2^)	0.42 ± 0.10	0.40 ± 0.18	0.28		
CFZ circularity	0.51 ± 0.08	0.58 ± 0.08	0.04	0.06	
Parafoveal SCP VD (%)	46.0 ± 3.20	49.1 ± 3.13	**< 0.01**	**< 0.01**	**2.9 (2.6–3.3)**
Parafoveal SCP VLD (%)	4.19 ± 0.29	4.45 ± 0.27	**0.03**	**0.04**	**1.7 (1.3–2.3)**
Parafoveal ICP VD (%)	44.1 ± 2.87	49.2 ± 3.10	**0.02**	**0.03**	**2.1 (1.8–2.4)**
Parafoveal ICP VLD (%)	4.65 ± 0.20	5.11 ± 0.26	**0.03**	0.12	
Parafoveal DCP VD (%)	43.4 ± 2.70	49.1 ± 3.42	**0.04**	0.11	
Parafoveal DCP VLD (%)	4.69 ± 0.24	4.99 ± 0.28	0.15		
Choriocapillaris FDD	42.31 ± 6.9	33.40 ± 6.4	**< 0.01**	**< 0.01**	**4.7 (4.2–5.0)**

BCVA = best-corrected visual acuity; CFZ = capillary free zone; CI = confidence interval; CMT = central macular thickness; D = diopters; DCP = deep capillary plexus; EZ = ellipsoid zone; FDD = flow deficit density; ICP = intermediate capillary plexus; LHEP = lamellar hole-associated epiretinal proliferation; OCTA = OCT angiography; OR = odds ratio; SCP = superficial capillary plexus; VD = vessel density; VLD = vessel length density.

Statistically signifiant values are highlighted in bold.

**Figure 2:**
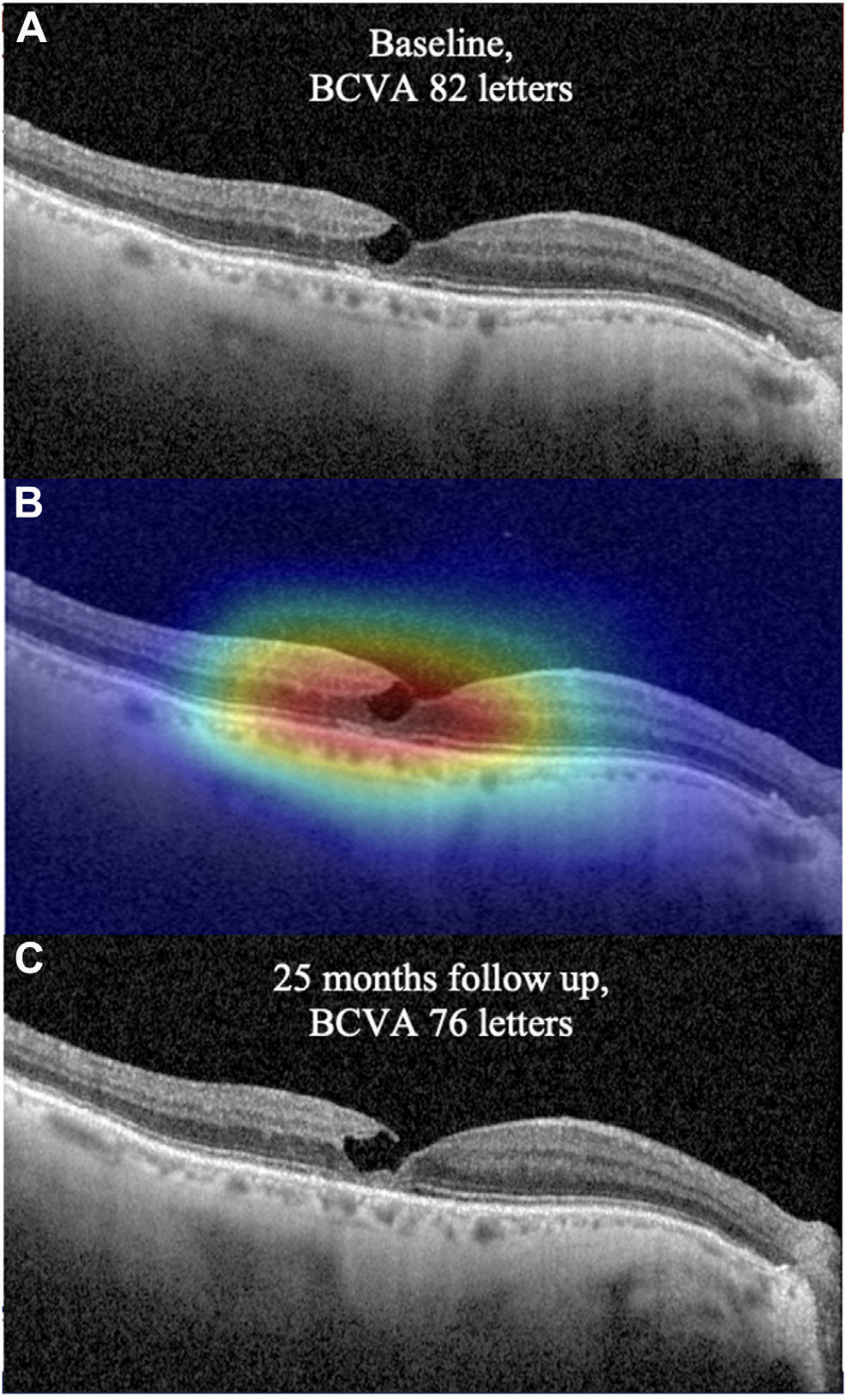
**A,** Baseline fovea crossing OCT B-scan acquisition of a LMH from the VA-PROG group. Parafoveal TL colocalizing with a zone of EZ disruption in the temporal fovea can be noted. **B,** GradCam visualization map highlighting the region that was fundamental to the software for classification in VA-PROG group. The hot area corresponds to critical OCT signs of VA progression according to the software. **C,** Follow-up acquisition demonstrating an anatomic progression of the TL and an increase in size of the EZ interruption accompanied by a VA loss of 6 ETDRS letters. BCVA = best-corrected visual acuity; EZ=ellipsoid zone; LMH= lamellar macular hole; TL = tissue loss; VA = visual acuity.

**Figure 3:**
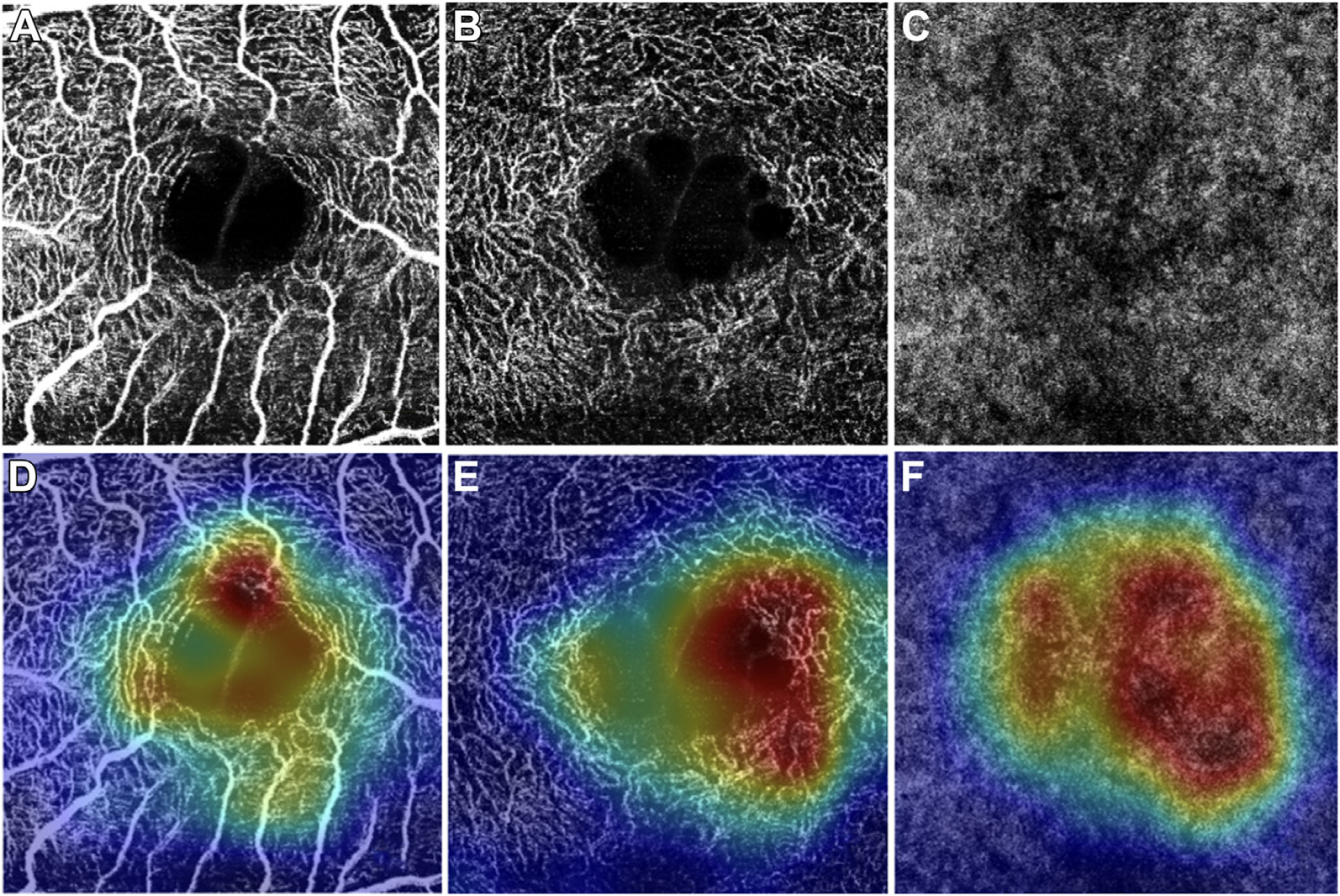
OCT angiogram of baseline SCP (**A**), DCP (**B**), and CC (**C**) with corresponding gradCAM maps (**D–F**) highlighting a zone of vascular rarefaction in a degenerative LMH experiencing VA loss during the follow-up. CC=choriocapillaris; DCP = deep capillary plexus; LMH = lamellar macular hole; SCP = superficial capillary plexus; VA = visual acuity.

GradCAM visualization method showed hot regions for functional progression located on zones of TL and EZ interruption in baseline OCT B-scan images (Fig 2) and on zones of parafoveal vascular rarefaction in OCTA images (Fig 3), including CC images.

## Discussion

Lamellar macular holes, which were originally considered quite static lesions, are indeed characterized by slow anatomic and functional changes, including both progression and regression of the lesion.^9^^,^^18^ Considering the potential complications of surgery (including the formation of iatrogenic FTMH^18^) and the high prevalence of stable LMHs, refinement of prediction of the natural course of each lesion would be beneficial to follow-up strategies and treatment decision making. The aim of our study was therefore to elaborate a DL model for prediction of progression of LMHs during a 2-year follow-up, from both an anatomic and functional point of view. Moreover, we aimed to identify biomarkers of anatomic and functional progression both with a classic method (regression analysis) and with an AI-based method. The DL model based on structural (OCT B-scan) and vascular (OCTA) imaging was able to predict VA loss of ≥5 ETDRS letters within 2 years of follow-up with an accuracy of 92.5%. A similar model could help predict natural course of LMHs, providing a good tool in both primary and tertiary care settings for refinement of follow up and treatment strategies. In particular, in LMHs characterized by a good VA and likely to show anatomic and functional stability over time, the risks of surgical treatment are not justified by potential benefits. Therefore, in these cases, indication for surgical treatment is weaker and should be carefully evaluated in each specific case. Notably, both OCT B-scan and OCTA information were combined to reach a high accuracy of the model, testifying to the importance of the microvascular component in the natural course of LMHs.

Relevant biomarkers predicting functional and anatomic progression were the presence of a EZ interruption, VPA at baseline, and TL within the first 6 months. This is consistent with previous findings from our group,^6^ which showed the speed of TL to be correlated with BCVA loss during time in LMHs. As a confirmation, areas of EZ interruption on baseline images and areas that were shown to be characterized by TL during the follow-up were identified in gradCAM maps as relevant areas for prediction on baseline images. The importance of EZ interruption in the natural history of LMHs was recently discussed by Chehaibou et al^18^ that described the development of an EZ interruption in around 30% of cases and a VA loss > 0.2 logarithm of the minimum angle of resolution to be more frequent in these cases. According to our findings, eyes experiencing VA deterioration are also characterized by a lower parafoveal SCP VD, VLD, and parafoveal ICP VD and a higher CC FDD compared with their stable counterpart. Considering the fact that TL involves prevalently the foveal area, parafoveal decrease in capillary blood flow should not be regarded as a mere reflection of the loss of foveal tissue. As a proof of that, this phenomenon involves also quadrants that are not affected by TL. Impairment of CC local blood flow is particularly interesting in this perspective, due to the possible physiopathologic correlation with EZ interruption. In fact, EZ damage in LMHs is often associated with retinal pigment epithelium damage,^19^ which could be regarded as the result of a reduction of CC blood flow providing metabolic exchange to the highly active retinal pigment epithelium cells. Other authors reported CC alterations in LMHs, even though an association with VA impairment in combination with other potential predictors has not been described yet.^22^^,^^23^ In a study from Kal et al,^23^ the authors found an increase in CC FDD in eyes affected by LMHs and theorized it to be the underlying mechanism to the development of EZ interruption in this type of lesion. Other authors postulated CC flow changes to precede the evolution of LMH into FTMH.^14^^,^^24^ In addition, secondary LMHs usually develop in diseases characterized by a reduction in CC and retinal plexuses’ blood flow such as age-related macular degeneration, MacTel,^25^^,^^26^ and myopia.^27^, ^28^, ^29^ As concerns SCP and ICP involvement, as previously described from our group, a possible explanation to flow reduction in the parafoveal area not involved by TL could be Muller-guided impairment in neurovascular unit function.^6^ AI-based assessment of biomarkers of VA loss performed in our study also confirmed the findings of the regression analysis concerning OCTA variables. In fact, not only did the SVM model including OCTA variables demonstrate high accuracy, but GradCAM visualization maps also highlighted the zones of vascular rarefaction in retinal capillary plexuses and CC as relevant regions for the classification task.

Baseline presence of epiretinal proliferation and baseline CMT were significant predictors of anatomic progression despite not being biomarkers of functional progression. Pang et al^30^ reported a lower CMT and EZ interruption to be associated with the presence of epiretinal proliferation. Interestingly, vitreopapillary adhesion was more strongly correlated with anatomic progression than with functional progression. The role of vitreopapillary adhesion is coherent with the findings from other authors demonstrating its relevance as a biomarker of progression in LMHs (*Invest Ophthalmol Vis Sci*. 59:5270, 2018).^4^ Lastly, baseline BCVA was lower in eyes characterized by progression of the lesion and the progression of the lesion was also correlated with age, consistent with previous reports in literature.^31^

In conclusion, to the best of our knowledge, we have provided the first demonstration of feasibility of automatic prediction of spontaneous functional progression of LMHs using a DL model. Moreover, we provide a comprehensive assessment of OCT B-scan and OCTA imaging predictors in a large population of idiopathic LMHs that we hope could help provide useful hints for further histologic studies concerning the pathogenesis and evolution of the disease. Limitations of our study include the relatively small sample size and the retrospective nature, which led to exclusion of several cases due to inhomogeneity of the acquisitions’ protocol. Moreover, the proposed software would need to be tested in a prospective protocol to confirm the good performance of the model in a real-life setting. On the other hand, the multicentric nature of the study makes the findings more likely to be trusted in clinical practice. Lastly, quantitative OCTA parameters have been demonstrated to vary significantly between images acquired with different devices and processed with different methods.^32^^,^^33^ For this reason, we recommend the use of the proposed DL tool in everyday practice only in case the acquisition device and the processing protocol used are coherent with what described in our article.
