# Supplementary material for: Prediction of Functional and Anatomic Progression in Lamellar Macular Holes
Source: Ophthalmol Sci. 2024 Apr 13;4(6):100529. doi: 10.1016/j.xops.2024.100529 (PMC11401036; doi:10.1016/j.xops.2024.100529)
Supplement: Supplementary File 1 [file mmc1.docx]

Scanning protocol for OCT B-scan consisted in:

- a radial scan centered on the papilla consisting in 6 sections with an angle of 30° and a length of 5.9 mm (20°), Automatic Real Time (ART) averaging was set to 9 frames. This scan was used to assess vitreopapillary adhesion (VPA).

- a 20°x20° (5.9 mmx5.9 mm) horizontal linear volume scan centered on the fovea with a 60 µm interval (98 sections) and an ART averaging set to 9 frames. A 60 μm interval is routinely used in the involved centers in the follow up of vitreoretinal interface diseases since it allows enface reconstruction of the vitreoretinal interface, improving the analysis of the evolution of the tangential tractions. The following acquisitions were considered for each raster to train the OCT B-scan based DL model: one fovea crossing scan, the first 2 scans superior to the fovea and the first 2 scans inferior to the fovea. As a result, 5 OCT B scan images for each eye were considered. This was done to avoid missing visual information coming from parafoveal regions.

Scanning protocol for OCTA was acquired using 85,000 A-scans per second using a light source centered at a wavelength of 840 nm (SD-OCTA), achieving optical axial resolution of 3.9 µm and transverse resolution of 5.7 µm. The 10°x10° protocol centered on the fovea was used. For each eye an enface OCTA scan from the superficial capillary plexus (SCP), intermediate capillary plexus (ICP), deep capillary plexus (DCP) and choriocapillaris (CC) were collected. OCT B scan and OCTA images were cropped using ImageJ software (National Institutes of Health, Bethesda, MD, USA).
