# Supplementary material for: Prediction of Functional and Anatomic Progression in Lamellar Macular Holes
Source: Ophthalmol Sci. 2024 Apr 13;4(6):100529. doi: 10.1016/j.xops.2024.100529 (PMC11401036; doi:10.1016/j.xops.2024.100529)
Supplement: Supplementary File 2 [file mmc2.docx]

Augmentation techniques included:

- random horizontal and vertical translations (both [-20/+20 pixels]) and random rotation on the frontal plane [-20°/+20°], to account for differences in head and eye position during the acquisition
- random rescaling [0.7/1.2], to account for axial length variability
- random reflection on the y axis for OCT B scan and both on the y and on the x axis for OCTA model, to account for differences in location of the tissue loss area

Adam optimization method was applied.^16^ Gradient-weighted Class Activation Mapping (GradCam)^17^ was used to highlight regions of the image that were decisive for final classification.
